# Drug Standardization through Pharmacognostic Approaches and Estimation of Anticancer Potential of Chamomile (*Matricaria chamomilla L.*) using Prostate-Cancer cell lines: An In-vitro Study

**DOI:** 10.7150/jca.77110

**Published:** 2023-02-05

**Authors:** Nida Khan, Mohd Afsahul Kalam, Mohd Tauseef Alam, Syed Anam Ul Haq, Wasia Showket, Zahoor A. Dar, Nida Rafiq, Waseem Mushtaq, Towseef Amin Rafeeqi, Mohammad Yunis Dar, Seema Akbar, Tariq Ahmad Butt, Riehana Gani, Uzma Majeed, Anis Ahmad Chaudhary, Hassan Ahmed Rudayni, Mohammed Al-Zharani, Sireen Abdul Rahim Shilbayeh, Ammena Yahia Binsaleh, Azza A. K. El-Sheikh, Zodwa Dlamini, Shabir Hussain Wani, Shahanavaj Khan, Khalid Z. Masoodi

**Affiliations:** 1 Department of Ilmul Advia (Pharmacology), RRIUM, University of Kashmir, Srinagar, J&K, India, 190006; 2 Department of Moalajat (Medicine), RRIUM, University of Kashmir, Srinagar, J&K, India, 190006; 3 Transcriptomics Laboratory (K-Lab), Division of Plant Biotechnology, Sher-e-Kashmir University of Agricultural Sciences and Technology of Kashmir, Shalimar, Srinagar, J&K, India, 190025; 4 Mountain Research Centre for Field Crops, Khudwani, Anantnag, 192101, Sher-e-Kashmir University of Agricultural Sciences and Technology of Kashmir, Srinagar, J&K, India, 192102; 5 Division of Forest Products & Utilization, Faculty of Forestry, Sher-e-Kashmir University of Agricultural Sciences and Technology of Kashmir, Benhama, Ganderbal, J&K, India 191202; 6 Allelopathy Laboratory, Department of Botany, Aligarh Muslim University, Aligarh, India 202002; 7 Biochemistry and Pathology Lab, RRIUM, Srinagar, University of Kashmir, J&K, India, 190006; 8 Phytochemistry lab, RRIUM, Srinagar, University of Kashmir, J&K, India, 190006; 9 SMPU Unit RRIUM, Srinagar, University of Kashmir, J&K, India, 190006; 10 Division of Agricultural Statistics, Sher-e-Kashmir University of Agricultural Sciences and Technology of Kashmir, Shalimar, Srinagar, J&K, India, 190025; 11 Department of Biology, College of Science, Al-Imam Muhammad Ibn Saud Islamic University, Riyadh, Saudi Arabia; 12 Department of Pharmacy Practice, College of Pharmacy, Princess Nourah bint Abdulrahman University, P.O. Box 84428, Riyadh 11671, Saudi Arabia; 13 Basic Health Sciences Department, College of Medicine, Princess Nourah bint Abdulrahman University, P.O. Box 84428, Riyadh 11671, Saudi Arabia; 14 SAMRC Precision Oncology Research Unit (PORU), DSI/NRF SARChI Chair in Precision Oncology and Cancer Prevention (POCP), Pan African Cancer Research Institute (PACRI), University of Pretoria, Hatfield 0028, South Africa; 15 Department of Biotechnology, University of Kashmir, Srinagar, J&K, India, 190006; 16 Department of Medical Lab Technology, Indian Institute of Health and Technology (IIHT), Deoband, Saharanpur, UP, India, 247554; 17 Department of Pharmaceutics, College of Pharmacy, King Saud University, Riyadh, Saudi Arabia, 11451

**Keywords:** *Gul-e-Babuna*, Anti-cancer, Antioxidant, *Saraṭān*, Prostate cancer, CFU, DPPH, TLC, phytomedicine

## Abstract

Cancer is the major challenge across world and the adenocarcinoma of prostate malignancy is the second most prevalent male cancer. Various medicinal plants are used for the treatment and management of various cancers. *Matricaria chamomilla* L., is one of the extensively used Unani medicament for the treatment of various type of diseases. In the current study we evaluated most of the parameters prescribed for drug standardization using pharmacognostic approaches. The 2,2 Diphenyl-1-picryl hydrazyl (DPPH) method was utilized for the analysis of antioxidant activity in the flower extracts of *M. chamomilla*. Moreover, we analyzed the antioxidant and cytotoxic activity of *M. chamomilla (Gul-e Babuna*) through in-vitro method. DPPH (2,2-diphenyl-1-picryl-hydrazl-hydrate) method was utilized for the analysis of antioxidant activity in the flower extracts of *M. chamomilla*. CFU and wound healing assay were performed to determine the anti-cancer activity. The results demonstrated that various extracts of *M. chamomilla* fulfilled most of the parameters of drug standardization and contained good antioxidant and anticancer activities. The ethyl acetate showed higher anticancer activity followed by aqueous, hydroalcoholic, petroleum benzene and methanol by CFU method. Also, the wound healing assay demonstrated that ethyl acetate extract has more significant effect followed by methanol and petroleum benzene extract on prostate cancer cell line (C4-2). The current study concluded that the extract of *M. chamomilla* flowers could act as good source of natural anti-cancer compounds.

## Introduction

The plant world has provided a never-ending supply of medicinal plants having diverse collection of biological traits and pharmacological uses. Plants have been utilized in many ways for thousands of years, including herbal teas, syrups, infusions, liniments, powder, and so on 1. According to the WHO, over 21,000 plant species can be utilized for medicinal approaches. Phytomedicine is a well-established and documented practice, and its applications are growing especially for balancing out the effects of contemporary lifestyle and diets. Herbs contain some of the most potent phytochemicals, that have been identified to have curative and preventive abilities synthesize secondary metabolites with various chemical structures including tannins, terpenoids, alkaloids, and flavonoids, which are involved in several therapeutic pharmacological effects due to presence of free radicals. Through various studies, it is found that polyphenols, flavonoids, alkaloids and terpenes present in the herbs act as antioxidant and anticancer agents 2. They provide nourishment for normal cell growth, repair, impede carcinogens, stimulate the immune system, and act as antioxidants, anti-ageing, and microbicidal agent 3, 4. German chamomile (*Matricaria chamomilla* L.) is a well-known specie of Asteraceae family sharing its origin from Mediterranean Basin and Eastern Europe and is grown all over the world. The scientific name "*Matricaria*" is derived from the Latin word matrix (uterus), referring to its tendency to relax uterine muscles related to mensus and postpartum abnormalities.

Medicinal plants are characterized in priority as an exhaustive source of bioactive compounds that are used in drug development 5, 6. Prostate cancer affects around one out of every nine men at some point in their lives, and in males prostate cancer is the major cause of mortality. In United States during year 2019, 33,330 deaths were estimated due to prostate cancer and 191,930 new cases were recorded 7. Males over the age of 50 and African American men are prone to prostate cancer. Out of 10 approximately 6 males above 65 are affected with this cancer, while males below forty are exceptionally uncommon. The average age at which a person is diagnosed is around 66 7, 8. There have been no published documents yet for confirming bioactive compounds from nutraceuticals and medicinal plants that have capability of lowering the nuclear localization of AR in CRPC cells directly and efficiently.

*M. chamomilla* is a vital drug in Unani medicine, and our study was carried out due to use it in the cancer treatment. Its effect on cancer may be due to its anti-inflammatory, demulcent, liquefier of the matters, and relaxant, diaphoretic and relaxant effect internally as well as externally by the mechanism of diversion and evacuation of matters causing the disease. Polyphenols' cytotoxicity on a variety of cancer cells has been established, and their antioxidant characteristics have been determined 9, 10. Purified flavonoids have demonstrated to have anticancer properties against hepatoma (Hep-G2), cervical carcinoma (Hela), and breast cancer in humans (MCF-7). Flavonoids suppress NF-B expression, which is important for cancer progression, angiogenesis, and proliferation 11. The most essential constituents of this medicine are, Flavonoids, Sesquiterpenes, Coumarins, Poly acetylenes, phenyl carboxylic acids, Mucins, Amino acids, Choline, Phytosterols, Mineral substances. In chamomile extract eleven bioactive compounds that include herniarin and umbelliferone (coumarin) and apigenin were identified. In *M. chamomilla* coumarins are delinated by presence of herniarin, umbelliferone and others 12, 13. So, the bioactive compounds present in *Babuna* (*M. chamomilla*) strengthen the hypothesis that this drug may be effective in the case of cancer due to its antioxidant property. The goal of this research was to assess the antioxidant and anti-malignancy activities of *M. chamomilla*. These goals may open new approaches to volarize sources of this plant species to fight against deadly diseases especially cancer.

## Materials and Methodology

### Plant Sample collection, identification and authentication

Test drug namely *Gul-e-Babuna* (*M. chamomilla*) flowers, were collected from the nearby market. The samples of plants were verified by the University of Kashmir's Center for Biodiversity and Taxonomy and were submitted to the museum of Centre for Biodiversity and Taxonomy under specimen voucher no.

### Parameters for drug standardization Macroscopic and organoleptic evaluation

Organoleptic evaluation of the procured samples was done to differentiate them from the related species having similar appearance. Parameters like shape, texture, colour, odour, taste were observed 14

### Microscopic evaluation

Microscopic evaluation aided by the stains allows a more detailed examination of the histological characters of the powdered drug for the correct identification. The flowers of *M. chamomilla* were crushed into powder form and boiled in solution of chloral-hydrate for 15-20 minutes. Small amount of powdered flower extract was observed under microscopic for various microscopic characters. Both stained and unstained slides were prepared. The stain used was phloroglucinol solution (1-2 drops of 0.1% W/V) and a drop of concentrated HCl to stain lignified cells pink 14.

### Physico-chemical evaluation

Certain Physico-chemical investigations including determination of ash values and extractive values were carried out for the formulations prepared 14, 15.

### Ash value

The proportion of inorganic substances present in a sample is determined by the ash value. The method was used to ascertain total ash, water-soluble ash and sulphated ash.

### Extractive values

The powder formulation of plant medication was air-dried and 5g of this powder was mashed using 100ml alcohol and water sequentially in a closed flask for 24 hours to determine extractive value. During the first 6 hours, the solutions were shaken repeatedly and left undisturbed for 18 hours. After that, filtration was done quickly to avoid solvent loss, followed by drying of, 25ml of filtrate at 50°C d through evaporation using a sunken dish having flat bottom to a consistent weight. At the end of this process, calculations for the alcohol percentage and water-soluble extract with reference to an air-dried drug were performed 15.

### Loss on drying

5 gm drug sample (in powdered form) was set on a sunken dish for evaporation at 105°C without being dried first, and the substance was weighed after 6 hours of drying. The drying procedure was continued until the difference between two consecutive weighing was less than 0.25 percent or two consecutive measurements were identical. Constant weight was achieved when the difference between two successive weights was less than 0.01g after drying for 30 minutes in a desiccator 16.

### Foaming index

One gram of Babuna flower powder was ground into a coarse powder and placed in a flask with 100 mL of boiling water. The concentrated liquid was made followed by filtration and was poured into 10 stopper vials (height 16 cm, diameter 16 mm) in increments of 1 ml, 2 ml, 3 ml, and so on, with level of liquid in all vials being corrected to 10 ml with water. After covering the test tube with a stopper, it was shaken for 15 seconds lengthwise. After resting the tubes upto 15 minutes, measurement of the foam height was recorded 15.

### Swelling index

One gram of finely ground and carefully weighed Babuna flowers were put in a stoppered measuring cylinder of 25 ml to which water was added at the rate of 25 ml, then mixture was vigorously jiggled at an interval of 10 minutes upto1 hour. The cylinder was left at room temperature for 3 hours before measurements were taken. Using 1g of plant material as a reference, the mean of the various readings was calculated 15.

### Fluorescence analysis

Many plants glow when cut surfaces or powders are subjected to Ultraviolet light, which may aid for their recognization. Fluorescence of the plant powder (40 mesh) was investigated in natural day light and under Ultraviolet light (254 and 366 nm), as well as after therapy with various chemicals such as picric acid, sodium hydroxide, acetic acid, ferric chloride, nitric acid, iodine, and hydrochloric acid 17.

### pH values

For the preparation of solutions, 1g and 10g of the accurately weighed drug was deliquesce in 100 ml of distilled water separately. Then the filtered extract was collected and pH values of solutions (1% and 10%) of *M. chamomilla* flowers were checked using a standardized pH meter and placed in tabulated form.

### Extraction of crude drug material

The dried and coarsely powdered material of Babuna (350gm) flowers were subjected to consecutive extraction in soxhlet extractor using different solvents in ascending order of their polarity e.g., petroleum benzene, ethyl acetate, methanol, hydro alcohol and aqueous. Extraction was performed using continuous hot percolation soxhlation 18.

### Preliminary phytochemicals screening of the extracts

The Pet. Benzene (PB), Ethyl acetate (EA), Methanol (MeOH), Hydro-alcohol (HA) and Aqueous (AQ) extracts of *Matricaria chamomilla* flowers were subjected to phytochemical screening. Phytochemical studies that were accomplished for identifying different constituents contained in the flowers.

### Test for alkaloids

Each extract (PB, EA, MeOH, HA and AQ) of the *M. chamomilla* flowers were assessed for the occurrence of alkaloids using Mayer's test, Tannic acid test, Wagner's test, Dragendroff's test and Hager's Test. Several grams of each extract were mixed in their respective solvents for preparing stock solution.

### Tests for glycosides

Each extract of the drug was tested for the availability of glycosides using Borntrager's test, Keller Killiani test and Legal's test.

A few grams of each extract were properly mixed in their respective solvents for preparing stock solution.

### Test for tannins

Each extract of the drug was tested for the occurrence of tannin using the Ferric chloride test and Lead acetate test. A few grams of each extract were properly mixed in their respective solvents for preparing stock solution.

### Test for carbohydrates

Each drug extract was tested for the availability of carbohydrates using Molisch's Test (for the presence of general sugars), Benedict's test (for reducing sugars), Fehling's test (for reducing sugars) and Barfoed's test (for reducing sugars).

### Test for flavonoids

Each drug extract was tested for the availability of flavonoids using the alkaline reagent test and Zinc test.

### Test for proteins

Each drug extract was tested for the availability of proteins using the Ninhydrin test and Millon's test.

### Test for saponins

Each drug extract was tested for the availability of saponins using the Lead acetate test, Froth test and Foam test.

### Test for terpenoids

Each drug extract was tested for the availability of terpenoids using the Salkowski test.

### Test for phytosterols

Each drug extract was tested for the availability of phytosterols using the Salkowski test.

### Thin Layer Chromatography (TLC)

Ethyl acetate and methanolic extracts from *M. chamomilla* were subjected to TLC profiling. The samples were soaked in appropriate solvents before being applied to TLC plates via capillary tubes. An appropriate solvent system was developed to act as a mobile phase for these extracts solutions. For ethyl acetate extract of *M. chamomilla* flower, the solvent system is made up of toluene, ethyl acetate and formic acid (3.5:3.5:1) respectively. The ethyl acetate decoction of *M. chamomilla* flower was implemented to a TLC plate using an appropriate capillary tube and established TLC plate, which then was independently placed in a TLC chamber for development using solvent system noted above as mobile phase. The TLC plate that was created was air-dried and then viewed in a UV chamber. Spots on the TLC plate were identified and the retention factor was calculated on the plate. The retention factor (R_f_) was calculated by the following method.



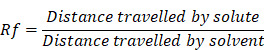



Similarly, Methanolic extract from *M. chamomilla* was treated with a distinct solvent system. The extract was applied on a TLC plate, which was then placed separately in a solvent system made up of toluene, ethyl acetate, formic acid (3:4:1) respectively. Spots on both TLC plates were identified and the retention factor was calculated on the plate by the method already mentioned 19.

### DPPH radical scavenging activity

According to the method described by Silva and Soysa the DPPH radical scavenging activity of the samples was decided, in which 950l of DPPH solution (100M in absolute methanol) was blended with 50l of varying extract concentrations (10, 50, 100, 150, 200, and 250g/ml). Further, the mixtures were shaken, followed by placing it in dark for 30 minutes and at 517 nm absorbance was measured 20, 21.

The radical scavenging activity was determined using below equation:



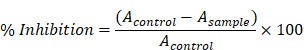



where, A_control_ represents the absorbance of control at t=0 min and A_sample_ represents the absorbance of the sample at t=30 mins. For refrence ascorbic acid was used as standard and for each test solution IC_50_ values were calculated i.e., the concentration essential for inhibiting the formation of 50% DPPH radical.

### Anti-cancer study

#### Cell lines

The C4-2 cell line of prostate cancer was procured from the University of Pittsburg, USA under MTA with the University of Chicago. C4-2 cell line was used in the current study as C4-2 cell line is highly cancerous and shows migration at a faster rate.

#### Methodology

The following procedures were utilized to test the materials (Medicinal plant, Cell line) used in the study:

#### Preparing crude plant extracts for cell culture

The dried and powdered flowers were subjected to the Soxhlet method of extraction with petroleum benzene, ethyl acetate, methanol, hydroalcoholic and aqueous solvents at their respective boiling temperatures for 48hrs. The extracts so obtained were dried in a rotatory vacuum evaporator (Perfit, India, Cat no. R300), further weighed and properly mixed in 10ml DMSO (Dimethyl sulphoxide), followed by filter sterilization using 0.2µm nylon filters (HiMedia). The extracts were formatted at a concentration of 12.5mg/ml and preserved in -20°C for rest of the analysis 22.

#### Cell line establishment

The RPMI media supplemented with 10% FBS, 1% L-glutamine and 100µg/ml penicillin-streptomycin was used for maintaining cell lines in 5% CO_2_ incubator at 37°C.

### Proliferation and migration studies

Proliferation and migration studies were conducted through Colony Formation Unit assay (CFU) and cell migration using wound healing assay 22.

Wound closure was calculated by using formula



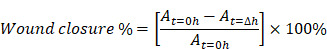




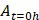
is the area of the wound measured immediately after scratching (t= 0 hour)


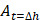
is the area of the wound measured h hours after the scratch is performed

### Statistical analysis

Graph Pad 7.0 Prism (Graph Pad, Inc. software) and MS (Microsoft) Excel 2007 were used for statistical analysis and diagrammatic construction. Data was represented as mean +/- SD and statistically significance was determined using ANOVA or Student's t-test as appropriate, three replications were used in the experiment. P values ​​of < 0.05 were referred as significant. CFU and wound healing assays were measured using Image J.

## Results

### Microscopic evaluation

**Fig 1:**
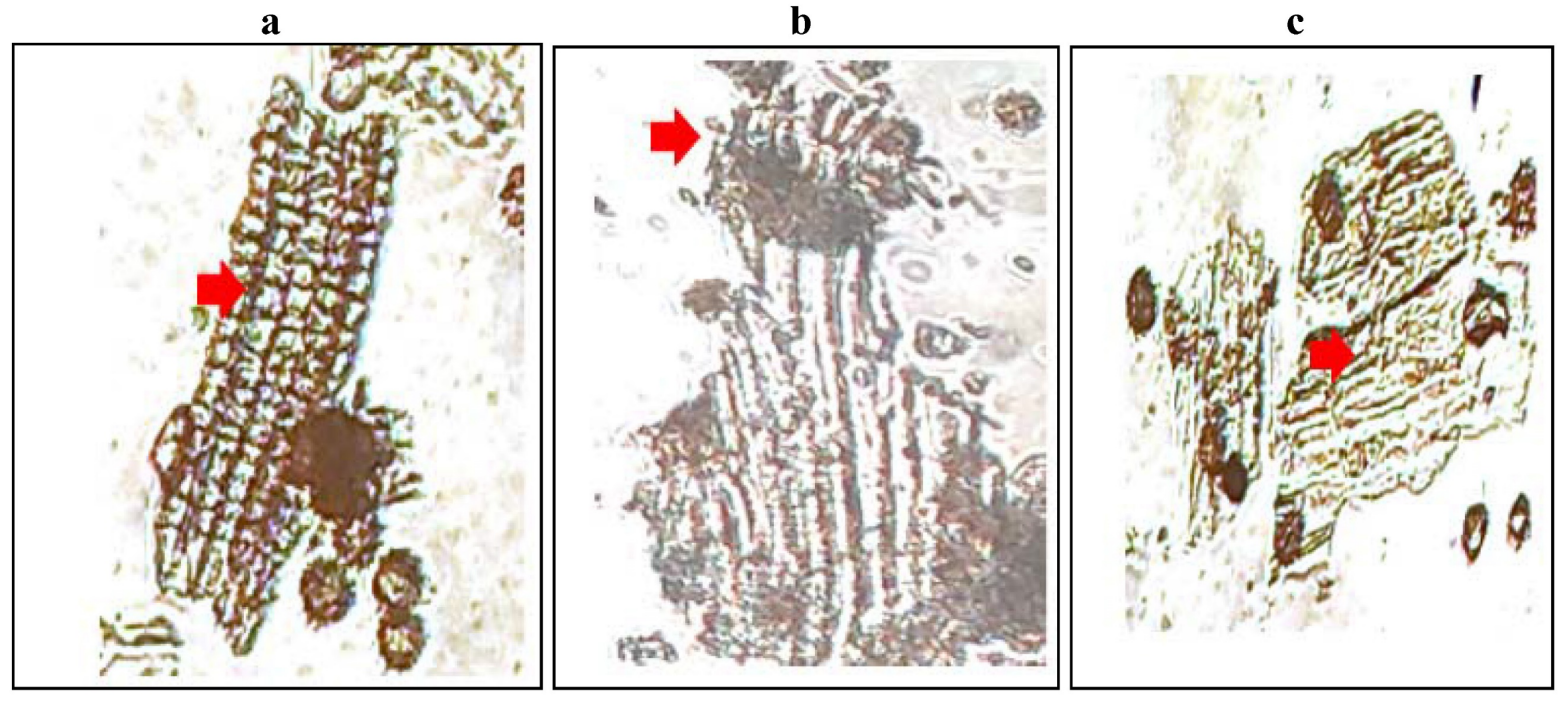
(a) Sclereids from the central region of a bract or palea. (b) Papillose stigma and part of the style in surface view with associated cluster crystals of calcium oxalate. (c) Outer epidermis near the base of the corolla in surface view.

The histological character of *M. chamomilla* flower powder is shown in Figure 1.

### Organoleptic evaluation

The organoleptic characters of dried flowers of *M. chamomilla* were evaluated and are tabulated in Table 1. From data, it was found that the dried flowers of Babuna were yellow-brownish in colour, moderately sour and specific fragrance and rough texture.

### Physicochemical parameters

#### Determination of ash values

The overall ash value of plant matter is reported in Table 2. Babuna had a total ash value of 61 percent, acid insoluble ash of 1.8 percent, and sulphated ash value of 6.4 percent, according to data obtained.

#### Determination of solvent extraction values

Polar elements such as phenols, alkaloids etc. in the plant sample is indicated by the ethanol-soluble extractive values. As shown in Table 2, the ethanol-soluble extractive values of Babuna flowers were 2.8 percent (hot extractive value) and 4.8 percent (cold extractive value). While as the water-soluble extractive values were found to be 11.8% and 15.8% as hot extractive value and cold extractive value respectively.

#### Losses due to drying

The data pertaining to losses due to drying is tabulated in Table 2. From data, it was found that there was a 6.8% loss on drying of Babuna flowers.

#### Determination of pH values

The pH values of Babuna flowers were found to be 5.6 and 5.52 for 1% and 10% solution respectively as shown in Table 2.

#### Determination of swelling and foaming index

The swelling and foaming index of dried flowers of Babuna were found to be 2 (swelling index) and <100 (foaming index).

#### Analysis for checking powdered drug fluorescence

Fluorescence characteristics of powder formulated drugs are present in Table 3 with various chemical reagents under visible and ultraviolet light.

#### Phytochemical screening

The analysis of phytochemicals of various solvents extracts of Babuna are tabulated in Table 4. The alkaloids were found in almost all extracts. Anthraquinone glycosides were present in only ethyl acetate, methanolic and hydro-alcoholic extracts while cardiac glycosides were present in only petroleum benzene, ethyl acetate and aqueous extracts. Tannins, carbohydrates, protein and saponins were absent in petroleum benzene extracts. Moreover, the aqueous extract also lacked the presence of tannins. Saponins were only present in hydroalcoholic and aqueous extracts. Moreover, terpenoids and sterols were present in all extracts.

#### Thin layer chromatography

**Fig 2:**
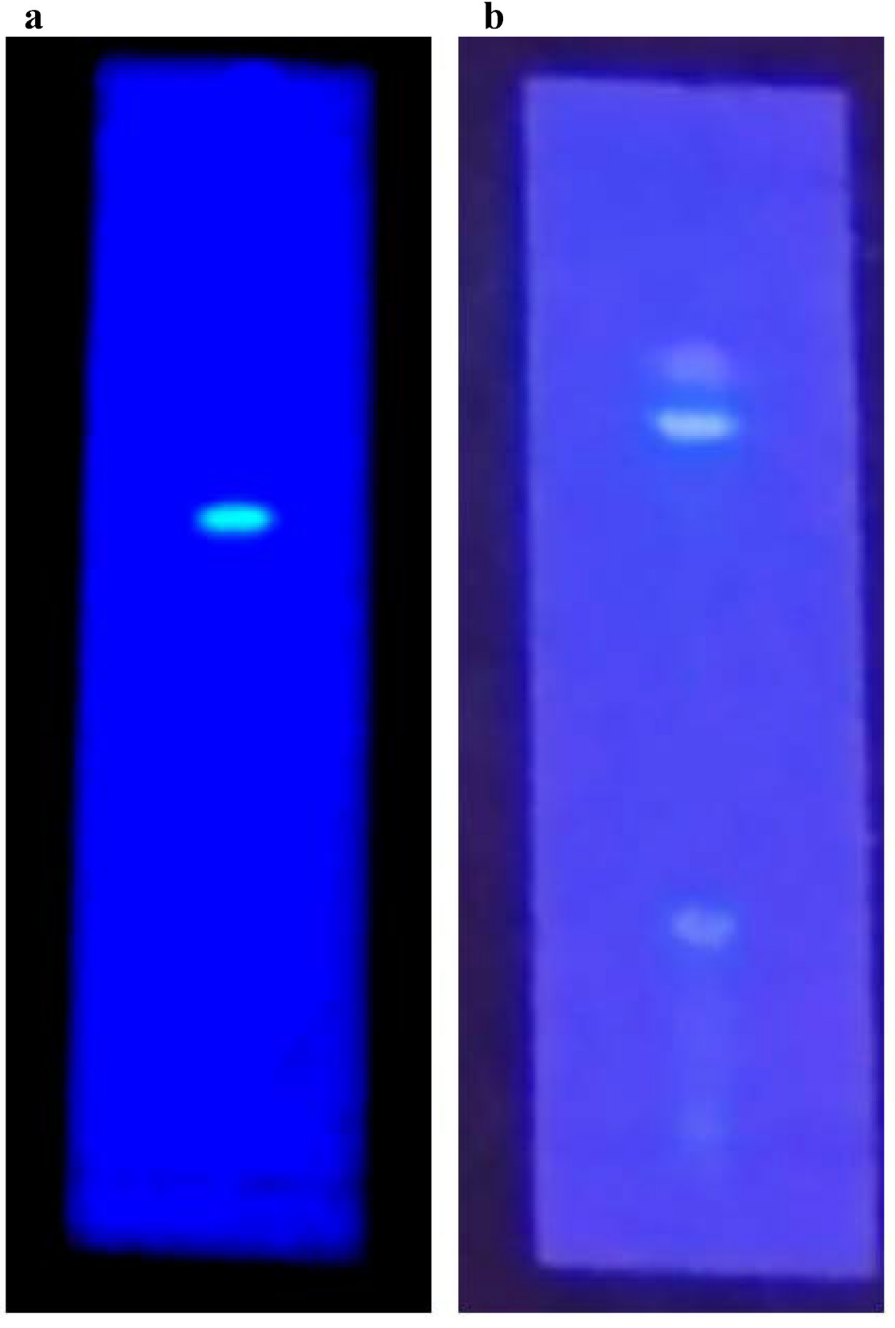
TLC profiling images of (a) Ethyl acetate extract. (b) Methanolic extract

TLC profiling of *Gul-e-Babuna* ethyl acetate extract in the Touluene: ethyl acetate: formic acid (3.5:3.5:1) solvent system indicated four bands with Rf values of 0.11, 0.51, 0.55, and 0.67. Thin layer chromatography of methanolic extract in Touluene: ethyl acetate: formic acid (3:4:1) solvent solution indicated existence of three bands with Rf values of 0.227, 0.606, and 0.636 (Table 5). Figure 2 shows TLC profiling pictures for various extracts (a, b).

#### DPPH radical scavenging activity

**Fig 3:**
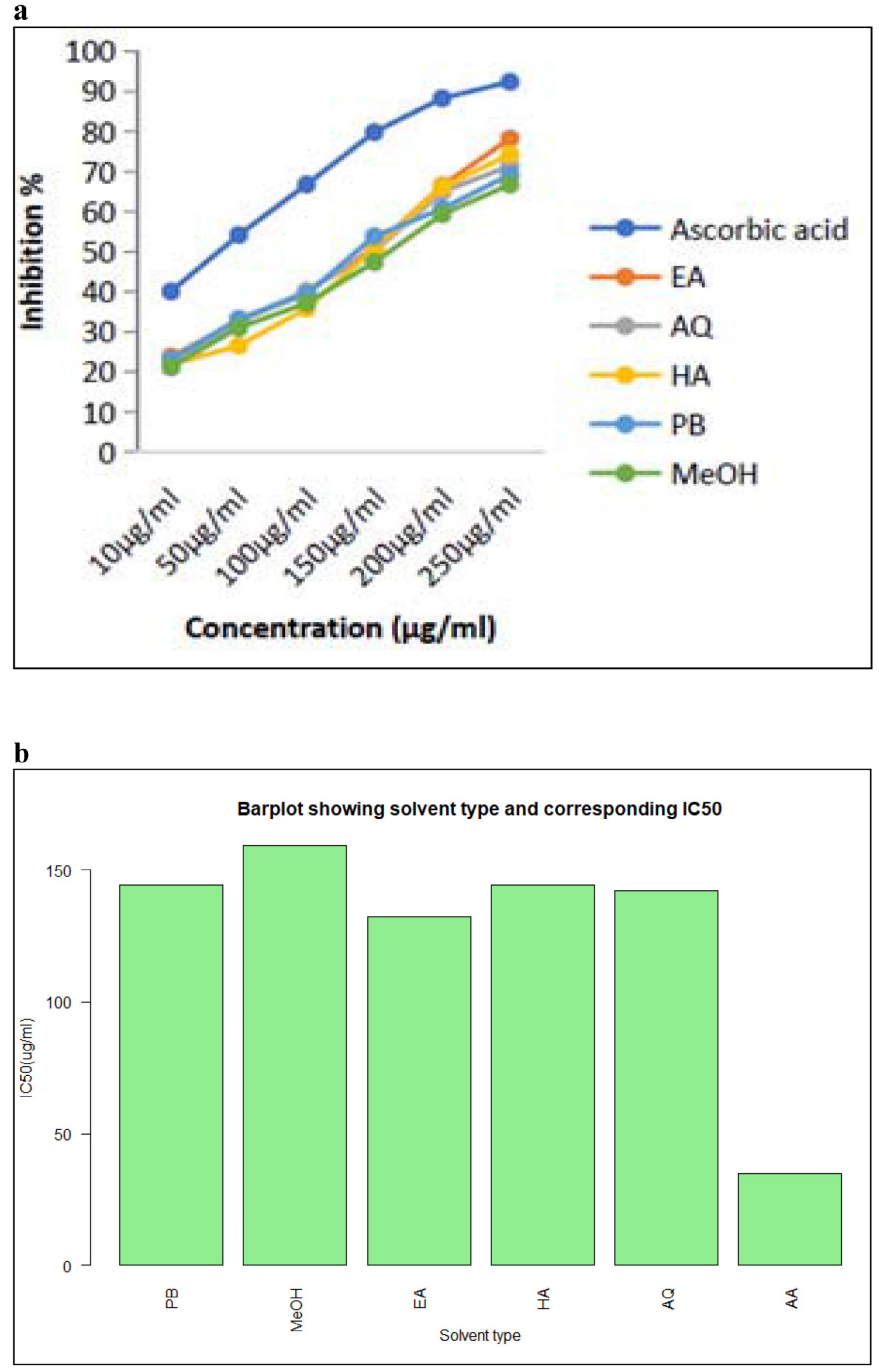

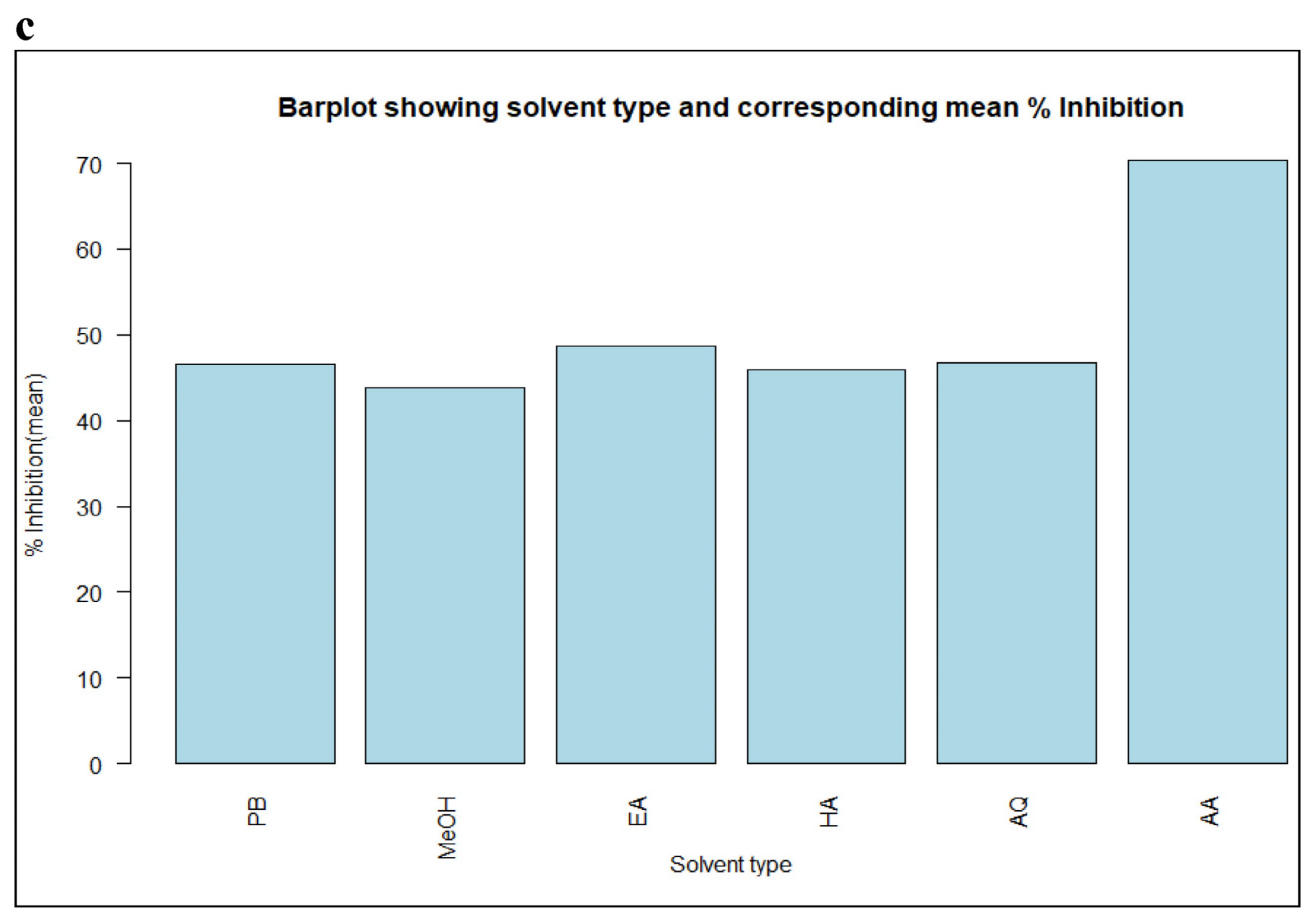
Graphical representation of (a) DPPH radical scavenging activity of Ascorbic acid, PB, EA, MeOH, HA and AQ extracts of *Matricaria chamomilla* L. flowers. (b) IC50 values of Ascorbic acid, PB, EA, MeOH, HA and AQ extracts of *Matricaria chamomilla* L.

The reduction in absorbance caused by plant antioxidants was used to test the DPPH radical scavenging activity of Petroleum benzene, ethyl acetate, methanol, aqueous, and hydroalcoholic extracts of *M. chamomilla* flowers. The inhibition of the DPPH free radical was obtained according to dose-dependent manner. The antioxidant activity of all extracts showed an increase as the concentrations increased. When the DPPH radical scavenging activity of *M. chamomilla* extracts were compared with respect to solvent, reduction ability was found to be much higher in ethyl acetate followed by aqueous extract and there was a slight difference in suppression among hydro alcoholic and petroleum benzene and finally methanolic extract with inhibition reaching up to 48.693 percent, 46.803 percent, 45.903 percent, 46.596 percent, 43.85 percent respectively (Table 6 and Figure 3a, b).

### Proliferation and Migration studies

#### Colony Forming Unit Assay (CFU)

**Fig 4:**
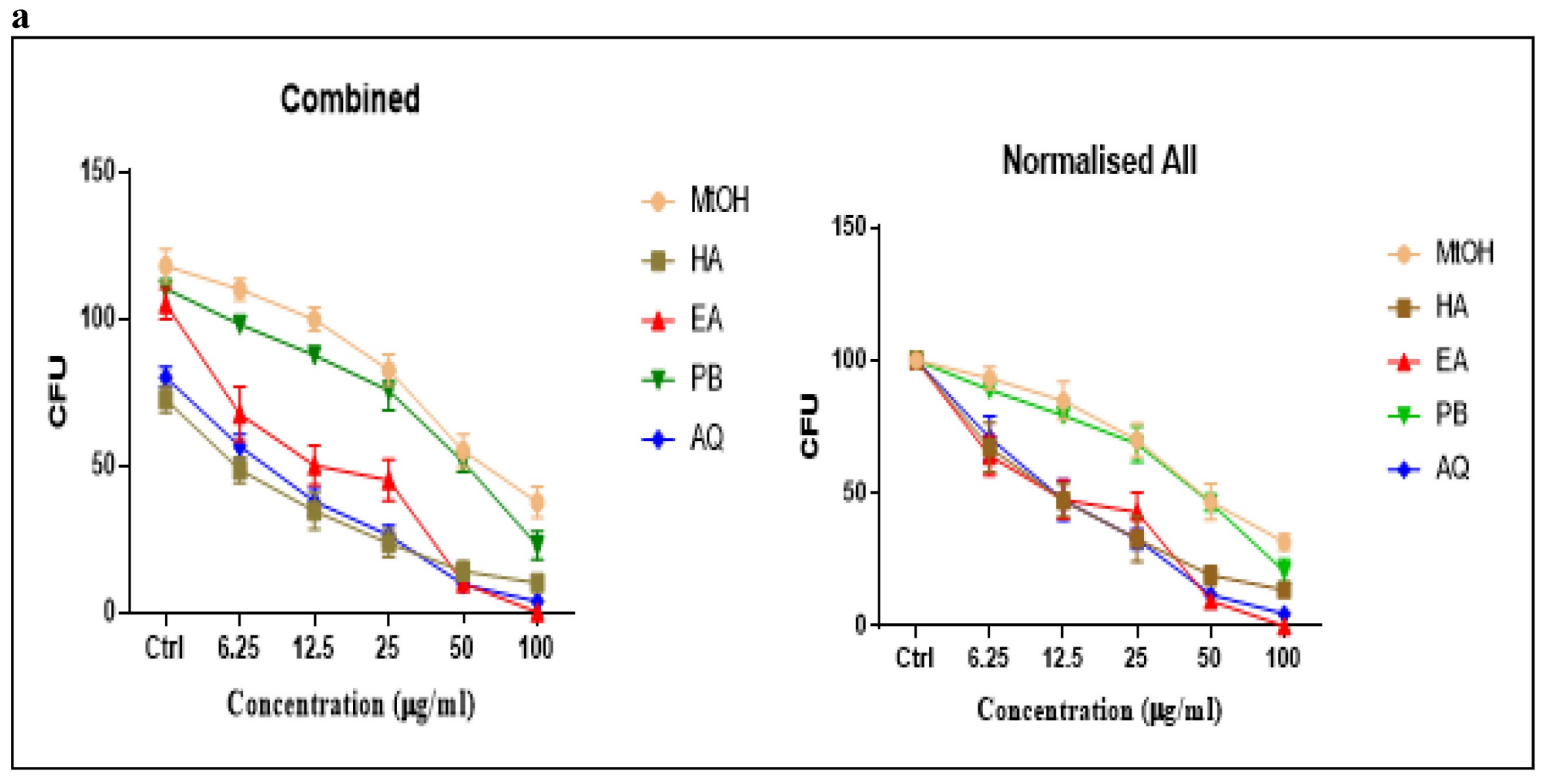

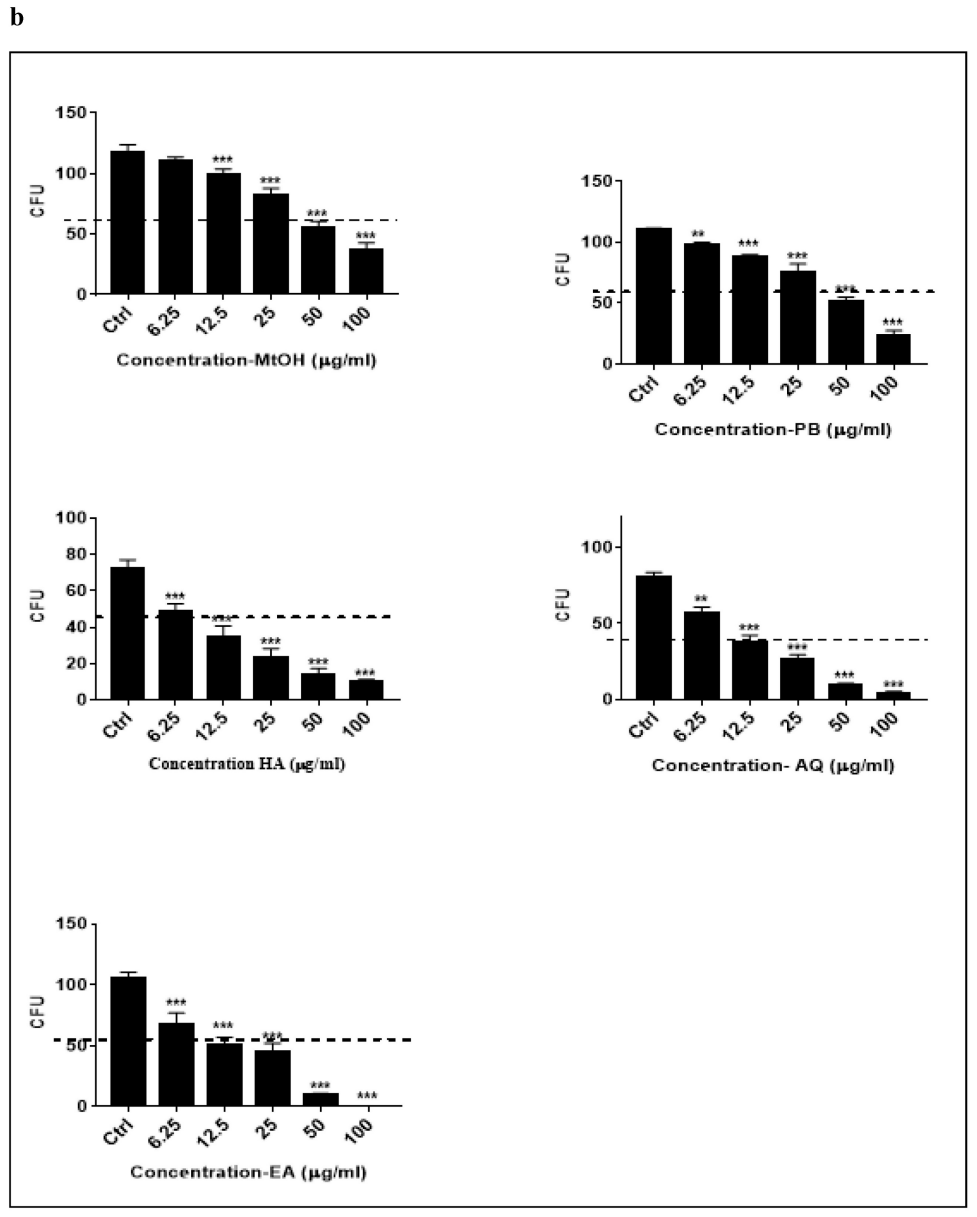
Dose-dependent inhibition of prostate cancer colony formation unit by of *Matricaria chamomilla* in C4-2 cells. (a) Effect of *Matricaria chamomilla* on inhibition of CFU in C4-2 cells by quantification of CFU. (b) Comparison of the effect of *M. chamomilla extracts* on inhibition of colony-forming units in C4-2 cells. Dashed line depicts IC50 value.

The plant extracts (Petroleum benzene, ethyl acetate, methanol, aqueous and hydroalcoholic) of *M. chamomilla* flowers inhibited colony-forming units with increasing concentration (6.25µg/ml, 12.5µg/ml, 25µg/ml, 50µg/ml, 100µg/ml). Ethyl acetate (p<0.001) showed best results in C4-2 cell line followed by aqueous extract (p<0.001) then hydro alcoholic (p<0.001), petroleum benzene (p<0.001) respectively and methanolic extract had less effect as compared to others as tabulated in Table 7. Comparison of the inhibition potential of five extracts (different concentrations) on CFU in C4-2 was observed in which ethyl acetate, aqueous extract and hydroalcoholic extracts showed more inhibition with increasing concentrations as shown in Figure 4 (a, b). Dunnett's multiple comparisons test for the effect of different extracts of *M. chamomilla* flower with control on inhibition of CFU in C4-2 cells is tabulated in Table 8.

#### Effect of *M. chamomilla* flower extracts on prostate cancer cells migration using wound healing assay

**Fig 5:**
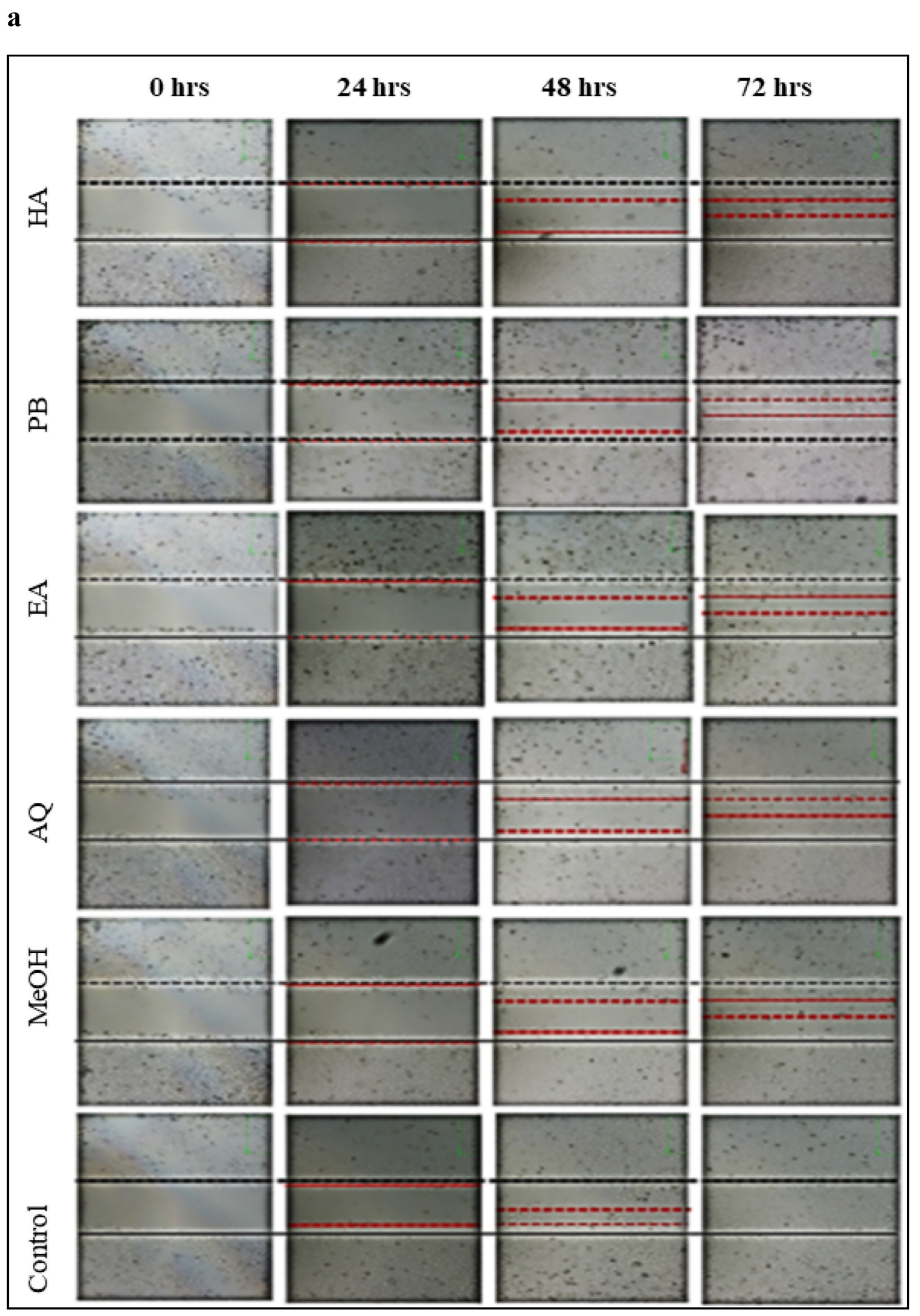

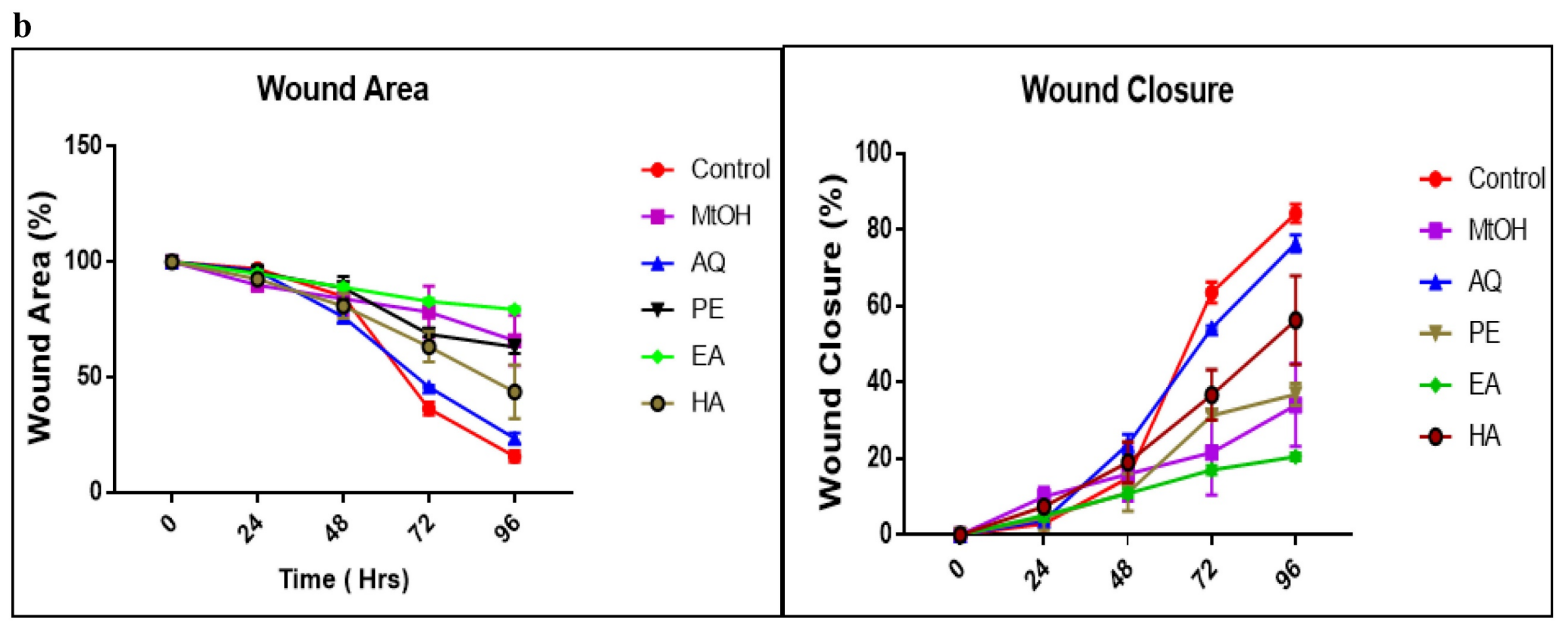
Inhibition of cellular migration/wound healing in C4-2 prostate cancer cells treated with *Matricaria chamomilla* (a) Confluent C4-2 cells were treated with (*Matricaria chamomilla*) extract (50µg/ml) after wounding across the cell monolayer with a sterile pipette tip and treated for 72hrs. Cells were imaged at 0, 24, 48, 72 hours after treatment and gap widths were measured. (b) Quantification of gap closure.

On comparing with control group as shown in Figure 5 (a,b), Babuna extracts reduced C4-2 tumor cell showed migration by 70 per-cent at a dosage of 50µg/ml. Gul-i-Babuna (*Matricaria chamomilla* L.) ethyl acetate extract produced significant results, followed by methanolic extract, with no significant difference between the effects of methanolic extract and petroleum benzene. Whereas the hydroalcoholic and aqueous extracts showed less effect as compared to others. Analysis of variance (ANOVA) in which three replications were carried out for inhibition of wound healing in C4-2 cells tested with plant extracts is tabulated in Table S1 and Dunnett's multiple comparisons test for inhibition of wound healing in C4-2 cells treated with flowers extracts of *M. chamomilla* is given in Table S2. In this test, at 72 hours all extracts showed a significant effect (p<0.001) except aqueous extract (p< 0.05) which showed less effect as compared to others. The results suggest that the extracts suppress tumor growth in cultured C4-2 cells and their effect does not promote prostate cancer cell migration. After 72 hours, there was no substantial wound healing in the treated cell line, whereas wound closure was close to complete in the untreated cell line (control) Figure 5 (a, b) Table S3-S4**.** The compound formulations of *M. chamomilla* ***viz*;** *Majoon-i-Falasfa, Ayarij-i-loghaziya, Majoon-i-Hafiz-ul- Ajsad, Raughan Samaat Kusha Jadeed, Qairooti-e-Babuna Wali, Qairooti-e-Arad-e-Baqla* with detailed ingredients used in these ingredients and their dosage and mode of action has been enlisted in Table 9.

## Discussion

Herbal medicines and their active ingredients have proved to be powerful medicaments against various cancers. Different plants are eaten up as functional foods as well as medicine, and medicine men, physicians, and scientists claim that they improve health. Medicinal plants such as *M. chamomilla, Melissa officinalis L.* [Lamiaceae]*, Taraxacum officinale* L. [Asteraceae]*, Hippophae rhamnoides* L. [Elaeagnaceae] may have great importance on human health by exerting antioxidant and anti-cancer effects 23, 24. Very little information on herbal medicines having a potential effect on prostate cancer is available. According to data, prostate cancer and breast cancer account for the majority of cancer cases in both men and women 22, 25. Various studies have been carried out on *M. chamomilla* like anti-inflammatory, antimicrobial, anti-anxiety, anti-oxidant but on prostate cancer, few studies were found 26, 27.

Due to their wide range of advantageous effects, many drug formulations and herbal tea of *M. chamomilla* source has gained extensive popularity. Nevertheless, the biochemical mechanisms involved for this plant's pharmacological efficacy are still unknown. The current research was carried out to explore the physicochemical parameters, phytochemical screening and *in vitro* antioxidant and anti-cancer effects present in *M. Chamomilla* flower extracts. For antioxidant activity, DPPH assay was used and for anti-cancer activity CFU and wound healing assays were used. The results demonstrate that plant extracts possess antioxidant and anticancer activities and this may provide a novel approach to prostate cancer therapeutic strategies.

### Antioxidant activity of chamomile extracts

The potential of essential oils to function as free radical scavengers was assessed using the change in absorbance caused by lower DPPH. The extracts' potential to inhibit the DPPH free radical was found to be concentration-dependent. The antioxidant activity of ethyl acetate, aqueous, hydroalcoholic, petroleum benzene, and finally methanol was found to be much higher, with IC50 values of 132, 141.901, 144.241, 144.34, and 159.077, respectively. The anti-free radical and antioxidant action of *Chamomile* extract (0.2-0.8 mg/mL) was discovered in experiments using sunflower oil as a model system 28.

### Anticancer activity of chamomile extracts

For anticancer activity CFU assay was used and the experiment was carried out on Prostate cancer cell line (C4-2) because this cell line is the most carcinogenic and shows high migration rates. Petroleum benzene, ethyl acetate, methanol, hydroalcoholic and aqueous extracts of* M. chamomilla* flowers were tested on C4-2 which showed a notable decrease in CFU's in a concentration-dependent manner. Ethyl acetate showed significant results in C4-2 cell line followed by aqueous extract then hydroalcoholic, petroleum benzene respectively and methanolic extract has less effect as compared to others. The active constituents of the extracts, that promote inhibition of cancer survival protein expression, are responsible for the decrease in CFUs. To verify the anti-migratory effect of the flower extract of *M. chamomilla* by the means of the scratch assay, the lowest concentration of 50µg/ml was tested on the C4-2 cell line. It can be observed in Figure 4 that, the extracts at the concentration of 50µg/ml, exhibited an anti-migratory effect on C4-2 cells. In this test at 72 hours, all extracts showed a significant effect (p<0.001) except the aqueous extract (p 0.05) that showed less effect as compared to others, p value range from 0 to 1, represents the statically significance of the results and probability of accepting or rejecting null hypothesis, ability of rejecting null hypothesis occurs and thus results are said to be statically significant. These results are in complete agreement with the studies that showed induction of cell growth inhibition and apoptosis when human prostate cancer PC-3 cells were exposure to aqueous and methanolic chamomile extract 29. Chamomile exerts selective dose-dependent cytotoxic response towards target cancer cells was also observed by other researchers 30. The major component of chamomile essential oil (αBisabolol), induced a reduction in cell proliferation and viability in pancreatic cancer cell lines (KLM1, KP4, Panc1, MIA, Paca2). A sesquiterpene (a-Bisabolol) present in chamomile has shown apoptosis in case of carcinoma cell line HepG2 in human liver 31.

Numerous bioactive constituents had been examined in countless medicinal plants due to their usefulness against different cancers such as colorectal cancers. The growth inhibitory activity of chamomile aqueous extract in virally transformed normal human prostate epithelium PZ-HPV-7 cells and other human prostate cancer cells, such as PC-3 and LNCaP cells, have been reported from multiple investigations. The viability of PZ-HPV-7 cells was reduced when they were exposed to chamomile 32. Another study found that natural compounds from *M. chamomilla* have anti-proliferative actions against MCF-7 in a dose-dependent manner 30. In pancreatic cancer cell lines (KP4, MIA, KLM1), α-Bisabolol, a significant component of chamomile essential oil, reduced cell proliferation and viability. The apoptotic impact of -Bisabolol, a sesquiterpene found in chamomile, on the human liver cancer cell line HepG2 was also discovered 33. Cell growth inhibition in several solid tumours and haematological malignancies by Apigenin has been shown. Its anti-cancer capabilities have been researched in vitro and in vivo extensively. It suppresses human prostate cancer and leukaemia development, arrests the cell cycle, and induces apoptosis through enhancing gap junction intercellular communication 13.

### Phytochemical evaluation

In the phytochemical evaluation, it was observed that *Matricaria chamomilla* L.flowers contain alkaloids, polyphenols (flavonoids and tannins), terpenoids, sterols etc. which can be the reason for cytotoxic and antioxidant properties in this wonderful herb. Flavonoids are primarily responsible for antioxidant and cytotoxic properties that help in protecting the cells from genetic mutation, oxidative damage, and eventually cancer. Many researchers have found that flavonoids suppress NF-B expression, which is important for cancer progression, angiogenesis, and proliferation. 11 thus, presence of flavonoids in this herb makes it a potential source for drug designing in various cancers. The ability of plant polyphenols to inhibit the growth of tumor cells due to interference with proteins found in tumor cells was also noted. Polyphenol can influence acetylation, methylation, or phosphorylation by interacting directly with cancer agents was reported by researchers 9, 10 thus favoring the exploration of *M. chamomilla* in cancer treatments.

## Conclusion

Based on the findings from the current study, the conclusion can be drawn that *M. chamomilla* plant extracts and formulations possess in vitro antioxidant and anticancer activities on prostate cancer cell line (C4-2) which might be useful in preventing oxidative stress during cancer treatment. The current study also validates the claims of Unani physicians that *Gul-e-Babuna* (*M. chamomilla*) can be used in prostate cancer treatment, but it needs further investigation and clinical studies for validation of anticancer effect in humans. In future, if phytoconstituents responsible for the anticancer effect on prostate cancer cell line are identified and isolated it could lead to the development of a novel natural remedy against prostate cancer. Therefore, Babuna flowers appear to be a rich source of a drug candidate that can restrict growth of prostate cancer cells. In a nutshell, the presented results support further investigation of Babuna flowers as an evolving remedial agent with high efficiencies for treatment of various cancerous conditions.

## Supplementary Material

Supplementary tables.

## Figures and Tables

**Table 1:** Macroscopic characters of dried flowers of *M. chamomilla* L

S. No	Parameters	*Matricariachamomilla* L. (Dried flowers)
1	Colour	Yellowish brown
2	Taste	Slightly bitter
3	Odour	Specific
4	Texture	Rough

**Table 2:** Physicochemical parameters of flowers of *M. chamomilla* L.

**Ash values**	**Particulars**		**Wt. of drug (gm)**	**Wt. of ash (gm)**	**% Yield of ash**
	Total ash value		5	3.05	61
	Acid insoluble ash value		5	0.09	1.8
	Sulphated ash value		5	0.32	6.4
**Solvent extractive values**	**Solvent**	**Type of extractive value**	**Wt. of drug (gm)**	**Wt. of dried extract (gm)**	**% Yield of extract (w/w)**
	Ethanol	Hot extractive value	5	0.28	2.8
		Cold extractive value	5	0.048	4.8
	Aqueous	Hot extractive value	5	0.118	11.8
		Cold extractive value	5	0.158	15.8
**Loss on drying**	**Part used**	**Wt. of drug (gm)**		**Loss on drying (gm)**	**% Loss on drying**
	Dried Flowers	5		0.068	6.8
**pH value**	**Sample**			**pH**	
	1% solution			5.6	
	10% solution			5.52	
**Swelling and foaming index**	**Sample**			**Swelling index**	**Foaming index**
	*Matricaria chamomilla* (Dried Flowers)			2	<100

**Table 3:** Fluorescence analysis of powdered drug with various chemical reagents under visible light, short and long wavelength.

Drug Treatment	Day light	UV (254nm)	UV (366nm)
Powder drug+ Distilled water	Light brown	Yellowish	Whitish
Powder drug + Conc. HCl	Yellowish green	Green	Dark brown
Powder drug +Dil. HCl	Transparent	Light yellow	Milky white
Powder drug + Conc. H_2_SO_4_	Dark brown	Brown	Milky white
Powder drug + Conc. HNO_3_	Light yellow	Yellowish green	Violet
Powder drug +chloroform	Transparent	Transparent	Violet
Powder drug + 10% NaOH	Yellow	Light green	Milky
Powder drug + picric acid	Yellow	Green	Black
Powder drug + Methanol	Transparent	Light green	Whitish
Powder drug + Ethyl acetate	Transparent	Light green	Transparent
Powder drug + glacial acetic acid	Transparent	Transparent	Light milky
Powder drug + Pet. Ether	Transparent	Light yellow	Brown
Powder drug + 10% Fecl3	Brown	Green	Black
Powder drug + Ammonia solution	Light green	Green	Milky white

**Table 4:** Phytochemical screening of Petroleum benzene, Ethyl acetate, Methanol, Hydro alcoholic, Aqueous extracts of flowers of *Matricaria chamomilla* L.

Phytochemicals	Tests	Petroleum benzene	Ethyl acetate	Methanol	Hydro-alcoholic	Aqueous
**Alkaloids**	Mayer's test	+ve	+ve	+ve	+ve	+ve
	Hager's test	+ve	+ve	+ve	+ve	-ve
	Wagner's test	+ve	+ve	+ve	+ve	-ve
	Dragendroff's test	+ve	+ve	+ve	+ve	+ve
**Anthraquinone glycosides**	Borntrager's test	-ve	+ve	+ve	+ve	-ve
**Cardiac glycosides**	Keller Killiani test.	+ve	+ve	-ve	-ve	+ve
	Legal's test	-ve	+ve	-ve	-ve	-ve
**Tannins**	Ferric chloride test	-ve	-ve	+ve	+ve	-ve
	Lead acetate test	-ve	+ve	+ve	+ve	-ve
**Carbohydrates**	Molisch's test	-ve	-ve	-ve	+ve	+ve
	Benedict's test	-ve	+ve	+ve	+ve	-ve
	Fehling's test	-ve	-ve	-ve	-ve	-ve
	Barford's test	-ve	-ve	-ve	-ve	-ve
**Flavonoids**	Alkaline reagent test	+ve	+ve	+ve	+ve	+ve
	Zinc test	-ve	+ve	+ve	+ve	-ve
**Proteins**	Ninhydrin test	-ve	+ve	-ve	+ve	-ve
	Millon's test	-ve	+ve	+ve	-ve	+ve
**Saponins**	Lead acetate test	-ve	-ve	-ve	-ve	-ve
	Froth test	-ve	-ve	-ve	+ve	+ve
	Foam test	-ve	-ve	-ve	-ve	-ve
**Terpenoids**	Salkowski test	+ve	+ve	+ve	+ve	+ve
**Sterols**	Salkowski test	+ve	+ve	+ve	+ve	+ve

**Table 5:** Thin Layer Chromatography (TLC) of EA and MeOH extracts of *M. chamomilla* flowers.

Extracts	Solvent system	Number of spots	R_f_ values	Remarks
Ethyl acetate	Touluene: ethyl acetate: formic acid (3.5:3.5:1)	4	0.11, 0.51, 0.55, 0.67	UV active
Methanol	Touluene: ethyl acetate: formic acid (3:4:1)	3	0.227, 0.606, 0.636	UV active

**Table 6:** DPPH based % inhibition and IC_50_ value of *Matricaria chamomilla* L. flower extracts using different solvents

	Concentration (µg/ml)							
Solvent type	% Inhibition						Mean	IC50 (µg/ml)
	10	50	100	150	200	250		
**Petroleum benzene**	23.09	33.23	39.39	53.87	60.75	69.25	**46.596**	**144.34**
**Methanolic extract**	21.29	31.06	37.19	47.40	59.37	66.79	**43.85**	**159.077**
**Ethyl acetate extract**	23.84	32.59	40.02	50.94	66.50	78.27	**48.693**	**132**
**Hydro alcoholic extract**	21.90	26.57	35.82	50.69	66.15	74.29	**45.903**	**144.241**
**Aqueous extract**	21.20	32.93	40.19	50.12	65.01	71.40	**46.803**	**141.901**
**Ascorbic acid (Standard)**	40.11	54.21	66.78	79.87	88.34	92.43	**70.29**	**34.755**

**Table 7:** Anti-cancer activity of different extracts of *Matricaria chamomilla* L. flowers by using CFU method

	Concentration (µg/ml)				
Solvent type	CFU quantification				
	6.25	12.5	25	50	100
**Petroleum benzene**	88.903 ± 2.265	79.371 ± 2.313	68.527 ± 6.801	46.478 ± 2.979	20.638 ± 4.431
**Ethyl acetate extract**	64.193 ± 7.361	47.720 ± 7.245	42.980 ± 7.561	9.560 ± 1.154	0 ± 0
**Methanolic extract**	93.329 ± 4.133	84.779 ± 7.136	70.072 ± 6.181	46.848 ± 6.814	31.675 ± 3.039
**Hydro alcoholic extract**	67.244 ± 9.328	47.371 ± 6.182	32.750 ± 8.516	19.187 ± 3.653	13.749 ± 1.302
**Aqueous extract**	70.886 ±8.181	47.397 ± 7.587	32.800 ± 3.737	11.944 ± 2.136	4.686 ± 2.056
**DMSO (Control)**	-	-	-	-	100 ± 0.0

**Table 8:** Dunnett's Multiple Comparison test

Extracts	Pair Comparison	Mean difference	Significance (P value)
PB	Ctrl vs 6.25	12.25	Yes (0.002)
	Ctrl vs 12.5	22.75	Yes (<0.001)
	Ctrl vs 25	34.75	Yes (<0.001)
	Ctrl vs 50	59	Yes (<0.001)
	Ctrl vs 100	87.5	Yes (<0.001)
EA	Ctrl vs 6.25	37.5	Yes (0.001)
	Ctrl vs 12.5	55	Yes (0.001)
	Ctrl vs 25	60	Yes (0.001)
	Ctrl vs 50	95	Yes (0.001)
	Ctrl vs 100	105	Yes (0.001)
	Ctrl vs 6.25	8	No (0.13)
	Ctrl vs 12.5	18.25	Yes (<0.001)
	Ctrl vs 25	35.5	Yes (<0.001)
	Ctrl vs 50	63	Yes (<0.001)
MeOH	Ctrl vs 100	80.5	Yes (<0.001)
	Ctrl vs 6.25	24	Yes (0.0001)
	Ctrl vs 12.5	38	Yes (0.0001)
	Ctrl vs 25	49	Yes (0.0001)
	Ctrl vs 50	58.5	Yes (0.0001)
HA	Ctrl vs 100	62.5	Yes (0.0001)
	Ctrl vs 6.25	23.5	Yes (<0.001)
	Ctrl vs 12.5	42.25	Yes (<0.001)
	Ctrl vs 25	53.75	Yes (<0.001)
	Ctrl vs 50	70.5	Yes (<0.001)
AQ	Ctrl vs 100	76.25	Yes (<0.001)

p <0.05, p<0.01, p<0.001, p>0.05 will be considered as significant, highly significant, extremely significant and insignificant respectively compared with control.

**Table 9:** Showing Compound formulations of Matricaria Chamomilla (*Babuna*) with their Ingredients. [ Arzani HMA, NFUM-II, NFUM-VI]

S. No	Compound Formulation	Ingredients used in compound formulation with their Dosage	Babuna Used as a	Action	Dosage
	***Majoon-i-Falasfa***	1. Zanjabeel 35g 2. Filfil 35g 3. Dar-i- Filfil 35g 4. Darchini 35g 5. Amla 35g 6. Post-i-Halela 35g 7. Shitraj Hindi 35g 8. Zarawand-i-Mudhiraj 35g 9. Khussiyatul Salab 35g 10. Maghz-i-chilghoza 35g 11.** Bekh-i-babuna 35g** 12. Narjeel 35g 13.** Gul-i-Babuna 28g** 14. Makveez-i-Munaqqa 105g 16. Shehad Musaffa Twice or Thrice of all drugs	1. Bekh-i-babuna 35g 2. Gul-i-Babuna 28g	1*. Falij* (Paralysis) 2. *Nisyan* (Dementia)	9-18g Orally
	***Ayarij-i-loghaziya***	1.** Babuna Pahadi 7g** 2. Zarawand Mudharaj 7g 3. Bahar Kunda Mushawwa 10.5g 4. Alwa 10.5g 5. Farfiyoon 10.5g 6. Zafran 10.5g 7. Juntiyana 10.5g 8. Fatrasaliyoon 10.5g 9. Ushaq 10.5g 10. Jawsheer 10.5g	Babuna 7g	1. *Dard-i-Khussiyatain* (Testicular Pain) 2. *Dard-i-Pusht* (Backache)	18g Orally
	***Majoon-i-Hafiz-ul-Ajsad***	1. Darchini 20g. 2. Post-e-Beikh Kibr 20g. 3. Bisfaij 20g. 4. Izkhar 20g. 5. Zafran 10g. 6. Sumbul-ut-Teeb 40 g. 7. Asaroon 15g. 8. Rewand Chini 15g. 9. Qust Shireen 15g. 10. Majeeth 15g. 11. Nagar Motha 15g. 12. **Raughan-e-Babuna 15ml.** 13. Qand Safaid 600g.	Roghan-e-Babuna 15ml	1.*Muhallil-i-Waram* (Anti-inflammatory) 2.*Mudirr-i-Bawl* (Diuretic)	5-10g Orally
	***Raughan Samaat Kusha Jadeed***	1. Marzanjosh 40g 2. Mako Khushk 40g 3. Lehsun 40g 4. **Gul-i-Babuna 40g** 5. Barg-e-Neeb 80g 6. Barg-e-Sukhdarshan 80g 7. Barg-e-Tambaku 20g 8. Kundur 20g 9. S indoor 10g 10. Raughan-e-Talkh 40ml 11. Sirka Naishkar 80ml	Gul-i-babuna 40g	1.*Muhallil* (Resolvent) 2.*Musakkin-i-Dard-i-Uzn* (Earache Sedative)	2 drops in each ear
	***Qairooti-e-Babuna Wali***	1. Gul-e-Banafsha 100g. 2. Iklil-e-Malik 100g. 3. **Babuna 100g.** 4. Aab Q.S. 5. Loab-e-Aspghol 50ml. 6. Loab-e-Gul-e-Khatmi 50ml. 7. Raughan-e-Badam Shireen Q.S. 8. Mom Safaid Q.S.	Babuna 100g	1.*Muhallil-i-Waram* (Anti-inflammatory) 2.*Musakkin* (Sedative)	Local application
	***Qairooti-e-Arad-e-Baqla***	1. Banafsha 100g. 2. Suboos-e-Gandum 100g. 3. Arad-e-Jau 100g. 4. Arad-e-Baqla 100g. 5. **Babuna 100g.** 6. Gul-i-Khatmi 100g. 7. Iklil-ul-Malik 100g. 8. Raughan-e-Mom 100ml. 9. Katan 100g. 10. Hulba 100g. 11. Aab-e-Karnab Q.S.	Babuna 100g	1.*Muhallil-i-Waram*	Local application

## Abbreviations

DPPH: 2,2 Diphenyl-1-picryl hydrazyl
CFU: Colony Forming Units
DMSO: Dimethyl Sulfoxide
TLC: Thin Layer Chromatography
ROS: Reactive Oxygen Species
GFP: Green Fluorescent Protein
AR: Androgen Receptor
