# Supplementary material for: Drug Standardization through Pharmacognostic Approaches and Estimation of Anticancer Potential of Chamomile (Matricaria chamomilla L.) using Prostate-Cancer cell lines: An In-vitro Study
Source: J Cancer. 2023 Feb 5;14(3):490–504. doi: 10.7150/jca.77110 (PMC9969581; doi:10.7150/jca.77110)
Supplement: Supplementary file 1 — Supplementary tables. [file jcav14p0490s1.pdf]

**Supplementary data:**

**Table S1:** Dunnett's multiple comparison test for the effect of petroleum benzene (PB) extract of *M. chamomilla* flower with control on inhibition of CFU in C4-2 cells

| **Dunnett's multiple comparisons test** | **Mean Diff.** | **95.00% CI of diff.** | **Significant** | **Summary** | **Adjusted P Value** |
|---|---|---|---|---|---|
| Ctrl vs. 6.25 | 12.25 | 4.573 to 19.93 | Yes | ** | 0.002 |
| Ctrl vs. 12.5 | 22.75 | 15.07 to 30.43 | Yes | *** | <0.001 |
| Ctrl vs. 25 | 34.75 | 27.07 to 42.43 | Yes | *** | <0.001 |
| Ctrl vs. 50 | 59 | 51.32 to 66.68 | Yes | *** | <0.001 |
| Ctrl vs. 100 | 87.5 | 79.82 to 95.18 | Yes | *** | <0.001 |

P<0.05, p<0.01, P<0.001, p>0.0.05 will be considered as significant, highly significant, extremely significant and insignificant respectively compared with control.

**Table S2:** Dunnett's multiple comparisons test for the effect of ethyl acetate (EA) extract of *M. chamomilla* flower with control on inhibition of CFU in C4-2 cells

| **Dunnett's multiple comparisons test** | **Mean Diff.** | **95.00% CI of diff.** | **Significant** | **Summary** | **Adjusted P Value** |
|---|---|---|---|---|---|
| Ctrl vs. 6.25 | 37.5 | 25.9 to 49.1 | Yes | **** | 0.0001 |
| Ctrl vs. 12.5 | 55 | 43.4 to 66.6 | Yes | **** | 0.0001 |
| Ctrl vs. 25 | 60 | 48.4 to 71.6 | Yes | **** | 0.0001 |
| Ctrl vs. 50 | 95 | 83.4 to 106.6 | Yes | **** | 0.0001 |
| Ctrl vs. 100 | 105 | 93.4 to 116.6 | Yes | **** | 0.0001 |

P<0.05, p<0.01, P<0.001, p>0.0.05 will be considered as significant, highly significant, extremely significant and insignificant respectively compared with control.

**Table S3:** Dunnett's multiple comparisons test for the effect of methanol (MeOH) extract of *M. chamomilla* flower with control on inhibition of CFU in C4-2 cells

| **Dunnett's multiple comparisons test** | **Mean Diff.** | **95.00% CI of diff.** | **Significant** | **Summary** | **Adjusted P Value** |
|---|---|---|---|---|---|
| Ctrl vs. 6.25 | 8 | -1.815 to 17.81 | No | Ns | 0.13 |
| Ctrl vs. 12.5 | 18.25 | 8.435 to 28.06 | Yes | *** | <0.001 |
| Ctrl vs. 25 | 35.5 | 25.69 to 45.31 | Yes | *** | <0.001 |
| Ctrl vs. 50 | 63 | 53.19 to 72.81 | Yes | *** | <0.001 |
| Ctrl vs. 100 | 80.5 | 70.69 to 90.31 | Yes | *** | <0.001 |

P<0.05, p<0.01, P<0.001, p>0.05 will be considered as significant, highly significant, extremely significant and insignificant respectively compared with control.

**Table S4:** Dunnett's multiple comparisons test for the effect of hydro alcoholic (HA) extract of *M. chamomilla* flower with control on inhibition of CFU in C4-2 cells

| **Dunnett's multiple comparisons test** | **Mean Diff.** | **95.00% CI of diff.** | **Significant** | **Summary** | **Adjusted P Value** |
|---|---|---|---|---|---|
| Ctrl vs. 6.25 | 24 | 15.46 to 32.54 | Yes | **** | 0.0001 |
| Ctrl vs. 12.5 | 38 | 29.46 to 46.54 | Yes | **** | 0.0001 |
| Ctrl vs. 25 | 49 | 40.46 to 57.54 | Yes | **** | 0.0001 |
| Ctrl vs. 50 | 58.5 | 49.96 to 67.04 | Yes | **** | 0.0001 |
| Ctrl vs. 100 | 62.5 | 53.96 to 71.04 | Yes | **** | 0.0001 |

P<0.05, p<0.01, P<0.001, p>0.0.05 will be considered as significant, highly significant, extremely significant and insignificant respectively compared with control.

**Table S5:** Dunnett's multiple comparisons test for the effect of aqueous (AQ) extract of *M. chamomilla* flower with control on inhibition of CFU in C4-2 cells

| **Dunnett's multiple comparisons test** | **Mean Diff.** | **95.00% CI of diff.** | **Significant** | **Summary** | **Adjusted P Value** |
|---|---|---|---|---|---|
| Ctrl vs. 6.25 | 23.5 | 16.95 to 30.05 | Yes | *** | <0.001 |
| Ctrl vs. 12.5 | 42.25 | 35.7 to 48.8 | Yes | *** | <0.001 |
| Ctrl vs. 25 | 53.75 | 47.2 to 60.3 | Yes | *** | <0.001 |
| Ctrl vs. 50 | 70.5 | 63.95 to 77.05 | Yes | *** | <0.001 |
| Ctrl vs. 100 | 76.25 | 69.7 to 82.8 | Yes | *** | <0.001 |

P<0.05, p<0.01, P<0.001, p>0.0.05 will be considered as significant, highly significant, extremely significant and insignificant respectively compared with control.

**Table S6:** Analysis of variance for inhibition of wound healing in C4-2 cells treated with *Matricaria chamomilla* Linn flowers.

| **Source of Variation** | **% of total variation** | **P value summary** | | **Significant** | **P value** |
|---|---|---|---|---|---|
| Interaction | 1.766 | Ns | | No | 0.8072 |
| Row Factor | 62.48 | **** | | Yes | <0.0001 |
| Column Factor | 28.8 | **** | | Yes | <0.0001 |
| **ANOVA table** | **SS** | **DF** | **MS** | **F (DFn, DFd)** | **P value** |
| Interaction | 909.9 | 25 | 36.4 | F (25, 72) = 0.7318 | P=0.8072 |
| Row Factor | 32188 | 5 | 6438 | F (5, 72) = 129.4 | P<0.0001 |
| Column Factor | 14835 | 5 | 2967 | F (5, 72) = 59.66 | P<0.0001 |
| Residual | 3581 | 72 | 49.73 | - | - |

P<0.05, p<0.01, P<0.001, p>0.05 will be considered as significant, highly significant, extremely significant and insignificant respectively compared with control.

**Table S7:** Dunnett's multiple comparisons test for inhibition of wound healing in C4-2 cells treated with *Matricaria chamomilla* L. flowers extracts

| Dunnett's multiple comparisons test | Mean Diff. | 95.00% CI of diff. | Significant | Summary | Adjusted P Value |
|---|---|---|---|---|---|
| **0** | | | | | |
| Control vs. MeOH | 0 | -9.334 to 9.334 | No | Ns | >0.99 |
| Control vs. AQ | 0 | -9.334 to 9.334 | No | Ns | >0.99 |
| Control vs. PE | 0 | -9.334 to 9.334 | No | Ns | >0.99 |
| Control vs. EA | 0 | -9.334 to 9.334 | No | Ns | >0.99 |
| Control vs. HA | 0 | -9.334 to 9.334 | No | Ns | >0.99 |
| **24** | | | | | |
| Control vs. MeOH | 7.108 | -2.226 to 16.44 | No | Ns | 0.19 |
| Control vs. AQ | 0.8085 | -8.526 to 10.14 | No | Ns | >0.99 |
| Control vs. PE | 1.584 | -7.75 to 10.92 | No | Ns | >0.99 |
| Control vs. EA | 2.142 | -7.192 to 11.48 | No | Ns | 0.96 |
| Control vs. HA | 4.505 | -4.829 to 13.84 | No | Ns | 0.60 |
| **48** | | | | | |
| Control vs. MeOH | 1.027 | -8.307 to 10.36 | No | Ns | >0.99 |
| Control vs. AQ | 8.886 | -0.4482 to 18.22 | No | Ns | 0.07 |
| Control vs. PE | -3.778 | -13.11 to 5.556 | No | Ns | 0.74 |
| Control vs. EA | -4.017 | -13.35 to 5.318 | No | Ns | 0.70 |
| Control vs. HA | 4.15 | -5.184 to 13.48 | No | Ns | 0.67 |
| **72** | | | | | |
| Control vs. MeOH | -41.91 | -51.24 to -32.57 | Yes | *** | <0.001 |
| Control vs. AQ | -9.389 | -18.72 to -0.05491 | Yes | * | 0.05 |
| Control vs. PE | -32.25 | -41.58 to -22.91 | Yes | *** | <0.001 |
| Control vs. EA | -46.5 | -55.83 to -37.16 | Yes | *** | <0.001 |
| Control vs. HA | -26.85 | -36.19 to -17.52 | Yes | *** | <0.001 |

**Table S8:** Effect of *M. chamomilla* L. flowers on cell migration in C4-2 cells (Wound closure)

| **Source of Variation** | **% of total variation** | **P value** | **P value summary** | **Significant** | |
|---|---|---|---|---|---|
| Interaction | 17.81 | <0.0001 | **** | Yes | |
| Row Factor | 67.69 | <0.0001 | **** | Yes | |
| Column Factor | 12.16 | <0.0001 | **** | Yes | |
| ANOVA table | SS | DF | MS | F (DFn, DFd) | P value |
| Interaction | 8950 | 20 | 447.5 | F (20, 60) = 22.84 | P<0.0001 |
| Row Factor | 34012 | 4 | 8503 | F (4, 60) = 434 | P<0.0001 |
| Column Factor | 6110 | 5 | 1222 | F (5, 60) = 62.37 | P<0.0001 |
| Residual | 1176 | 60 | 19.59 | | |

**Table S9:** Dunnett's multiple comparison test on wound closure in C4-2 cells treated with *Matricaria chamomilla* L. flowers extracts

| Dunnett's multiple comparisons test | Mean Diff. | 95.00% CI of diff. | Significant | Summary | Adjusted P Value |
|---|---|---|---|---|---|
| **0** | | | | | |
| Control vs. MeOH | 0 | -9.334 to 9.334 | No | Ns | 0.9999 |
| Control vs. AQ | 0 | -9.334 to 9.334 | No | Ns | 0.9999 |
| Control vs. PE | 0 | -9.334 to 9.334 | No | Ns | 0.9999 |
| Control vs. EA | 0 | -9.334 to 9.334 | No | Ns | 0.9999 |
| Control vs. HA | 0 | -9.334 to 9.334 | No | Ns | 0.9999 |
| **24** | | | | | |
| Control vs. MeOH | -7.108 | -16.44 to 2.226 | No | Ns | 0.1924 |
| Control vs. AQ | -0.8085 | -10.14 to 8.526 | No | Ns | 0.9997 |
| Control vs. PE | -1.584 | -10.92 to 7.75 | No | Ns | 0.9905 |
| Control vs. EA | -2.142 | -11.48 to 7.192 | No | Ns | 0.9646 |
| Control vs. HA | -4.505 | -13.84 to 4.829 | No | Ns | 0.6020 |
| **48** | | | | | |
| Control vs. MeOH | -1.027 | -10.36 to 8.307 | No | Ns | 0.9984 |
| Control vs. AQ | -8.886 | -18.22 to 0.4482 | No | Ns | 0.0672 |
| Control vs. PE | 3.778 | -5.556 to 13.11 | No | Ns | 0.7440 |
| Control vs. EA | 4.017 | -5.318 to 13.35 | No | Ns | 0.6982 |
| Control vs. HA | -4.15 | -13.48 to 5.184 | No | Ns | 0.6721 |
| **72** | | | | | |
| Control vs. MeOH | 41.91 | 32.57 to 51.24 | Yes | **** | 0.0001 |
| Control vs. AQ | 9.389 | 0.05491 to 18.72 | Yes | * | 0.0482 |
| Control vs. PE | 32.25 | 22.91 to 41.58 | Yes | **** | 0.0001 |
| Control vs. EA | 46.5 | 37.16 to 55.83 | Yes | **** | 0.0001 |
| Control vs. HA | 26.85 | 17.52 to 36.19 | Yes | **** | 0.0001 |

$P<0.05$, $p<0.01$, $P<0.001$, $p>0.05$ will be considered as significant, highly significant, extremely significant and insignificant respectively compared with control. Data was statistically analyzed by ANOVA followed by Dunnett's test given in Table 19 and 20.
